# Cell orientation characteristics of the natural combs of honey bee colonies

**DOI:** 10.1371/journal.pone.0263249

**Published:** 2022-02-07

**Authors:** Shunhua Yang, Qingxin Meng, Wenzheng Zhao, Jianming Wang, Yiqiu Liu, Xueyang Gong, Kun Dong

**Affiliations:** Yunnan Provincial Engineering and Research Center for Sustainable Utilization of Honeybee Resources, Eastern Bee Research Institute, College of Animal Science and Technology, Yunnan Agricultural University, Kunming, China; King Khalid University, SAUDI ARABIA

## Abstract

The cell orientation characteristics of the natural combs of honey bees have received much research attention. Although natural combs have been shown to be composed of cells with three orientations—vertical, intermediate (oblique), and horizontal—the proportion of comb cells in these three orientations varies. Knowledge of the comb-building preferences of honey bees is essential for the installation of wax comb foundations, and clarification of the cell orientation characteristics of natural honey bee combs is important for beekeeping. The purpose of this study was to determine the cell orientation characteristics of natural combs of Eastern honey bees (*Apis cerana cerana*) and Western honey bees (*Apis mellifera ligustica*). Newly built combs were used to measure the orientation of hexagonal cells and calculate the proportion of cells in different orientations relative to the total number of cells. The number of eggs laid by queens in the cells of different orientations was also determined. The orientation of cells in the natural combs of Eastern and Western honey bees was determined based on the value of the minimum included angle between the pair of parallel cell walls and a vertical line connecting the top and bottom bars of the movable frame in the geometric plane of the comb: 0°≤θ≤10°, 10°<θ≤20°, and 20°<θ≤30° for vertical, intermediate, and horizontal orientations, respectively. Natural combs were composed of cells with at least one orientation (vertical or horizontal), two orientations (vertical + intermediate (oblique) or vertical + horizontal), or three orientations (vertical + intermediate + horizontal), and the proportions of combs with the three aforementioned configurations differed. Both Eastern honey bees and Western honey bees preferred building combs with cells in a vertical orientation. Queens showed no clear preference for laying eggs in cells of specific orientations. The results of this study provide new insight that could aid the production and cutting of wax comb foundations of Eastern and Western honey bees. Our study highlights the importance of installing wax comb foundations compatible with the comb-building preferences of bees.

## 1 Introduction

In 1857, Johannes Mehring invented the flat plate foundation press, and this tool has since been used widely to produce wax comb foundations for beekeeping and research [1, 2]. The foundation press has the cell base structure of natural honey bee combs engraved on the surface of a flat plate that can be used to print beeswax sheets with the cell base structure of natural honey bee combs (i.e., comb foundation). The beekeeper places the wax comb foundation into a movable frame, which worker bees then use to build the cell wall and form the comb.

**Fig 1:**
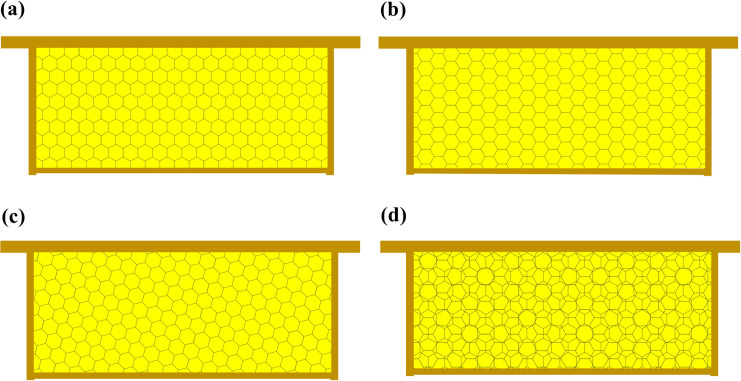
Schematic of combs with cells in different arrangements and orientations. Combs with cells that are (a) oriented vertically, (b) oriented horizontally, (c) oriented obliquely, and (d) arranged in a rosette pattern (i.e., cells on one side of the comb are oriented vertically, whereas cells on the other side of the comb are oriented horizontally).

One issue requiring careful consideration in beekeeping is the orientation of comb cells, specifically that of the cell base structure of the wax comb foundation when it is embedded in movable frames. Digges [3] specified two types of orientations: vertical and horizontal. In a vertical orientation, cells have pairs of parallel cell walls that are perpendicular to the top and bottom bars of the movable frame (Fig 1A). In a horizontal orientation, cells have pairs of parallel cell walls that run parallel to the top and bottom bars of the movable frame (Fig 1B). Allen [4] indicated that honey bees can build combs with cells with both vertical and horizontal orientations. Some researchers later proposed that an intermediate (oblique) orientation, in which all cell walls of the comb are neither perpendicular nor parallel to the top and bottom bars of the movable frame (Fig 1C), would result in the construction of superior combs [5].

Honey bee combs are two-sided structures, and in the vertical, intermediate, and horizontal orientations [5, 6], the cells on the two sides of the comb have the same orientation. However, the cells can also be arranged in a rosette pattern, wherein the cells on the two sides of the comb differ in their orientation (Fig 1D) [7–9]. For example, the cells on one side of the comb can be oriented vertically, and the cells on the other side of the comb can be oriented horizontally. There are three possible cell rosette patterns in theory: vertical + horizontal, vertical + intermediate, and horizontal + intermediate.

Many observations have been made of natural honey bee (*Apis mellifera ligustica*) combs and natural nests. Allen [4] noted that bees tended to build combs with cells in a horizontal orientation when the bees were not provided with wax comb foundation. Betts [5] reported two contrasting observations. One experienced beekeeper indicated that one-third of natural combs have horizontal cells, and two-thirds of natural combs have vertical cells. However, A. Podolski only detected combs with cells in an intermediate orientation—never combs with cells oriented vertically or horizontally. Thompson [6] made observations of 268 natural combs and found that the proportion of combs with cells in a vertical orientation was 48.88% (n = 131), the proportion of combs with cells in a horizontal orientation was 45.90% (n = 123), the proportion of combs with cells in an intermediate orientation was 4.85% (n = 13), and the proportion of combs with cells in vertical and horizontal orientations was 0.37% (n = 1). Another study showed that the proportion of combs with cells in a vertical orientation was 46%, the proportion of combs with cells in a horizontal orientation was 29%, and the proportion of combs with cells in an intermediate orientation was 25% [10]. Lastly, Yang et al. [11] found that the proportion of combs with cells in a vertical orientation was 83% and that the proportion of combs with cells in vertical and horizontal orientations was 17%.

The orientation of the brood cells from which honey bee workers emerge might determine the orientation of newly built cells. However, the results of previous studies do not support this hypothesis: honey bees emerging from cells with a vertical orientation can build new cells in either vertical or intermediate orientations, and honey bees emerging from cells in a horizontal orientation can build new cells in either vertical, intermediate, or horizontal orientations [9]. These findings indicate that the orientation of brood cells is not the main factor determining the orientation of newly built cells. Nevertheless, the orientation of the newly built cells has often been noted to follow the orientation of already existing cells [12], including the orientation of the cell base of the wax comb foundation provided by beekeepers or researchers. Therefore, the orientation of the original cells of the comb might be the main factor affecting the orientation of newly built cells. The orientation of newly built cells can also be affected by the previous experience of honey bees [13, 14]. Another factor that has been speculated to affect the orientation of cells is gravity. However, under both zero-gravity conditions and natural conditions, honey bees are capable of building natural comb cells in vertical, horizontal, and intermediate orientations [15], suggesting that gravity does not play a major role in guiding the orientation of cells. Pratt [16] showed that there was a significant correlation between the orientation of cells and substrate when bees were induced to build comb on substrates. In sum, the orientation of the cells of natural combs is not affected by the orientation of the brood cells from which workers emerge and gravity. Instead, previous work suggests that the most important factors affecting the orientation of cells include the prior experience of bees in building cells and the orientation of the substrate.

Wax comb foundations are widely used in modern beekeeping production. When beekeepers place wax comb foundations into movable frames that the bees use to build combs, the orientation of the cell base structure of the wax comb foundation should be based on the preferences of the honey bees. Previous studies of the natural combs of Western honey bees (*A*. *mellifera ligustica*) (Hymenoptera: Apidae) have revealed wide variation in cell orientation, and the cell orientation of natural combs of Eastern honey bees (*Apis cerana cerana*) (Hymenoptera: Apidae) has not yet been studied. The aim of this paper was to determine the cell orientation characteristics of natural combs of Eastern honey bees and Western honey bees as well as whether these species show preferences for constructing combs with specific cell orientations. We (1) made quantitative observations of the orientation of the cells of natural combs and counted the number of cells with different orientations; (2) calculated the proportion of cells in different orientations (vertical, intermediate, and horizontal); (3) calculated the percentage of combs with cells in a rosette arrangement relative to the total number of combs; and (4) compared the number of eggs laid by queens in comb cells in vertical, intermediate, and horizontal orientations to assess oviposition preferences. Generally, the results of this study provide valuable information that can be used to aid the production of wax comb foundations for Eastern and Western honey bees.

## 2 Materials and methods

### 2.1 Newly built natural combs

This study was conducted in the apiary of Yunnan Agricultural University, which is located in Kunming, Yunnan Province, China, from March to August 2021. Colonies of Eastern and Western honey bees were set up in standard movable-frame Langstroth hives. There were 38 colonies of Eastern honey bees, and four frames were fully covered by adult bees. There were 27 colonies of Western honey bees, and seven frames were fully covered by adult bees. In both sets of colonies, queens were of the same age, and colonies were of similar strength.

The hives of each colony were divided into two areas by a vertical queen excluder. Each area in the Eastern honey beehives had two wax combs. The Eastern honey bee queen laid eggs in one of two areas, and an empty movable frame was added into the other area so that worker bees could build natural comb. There were three combs in one area of the Western honey beehives for rearing broods, and there were four combs in another area where an empty frame was added so that worker bees could build natural combs.

Each colony was fed with sugar syrup every evening during comb-building until the inner area of the empty frame was completely occupied by wax comb. The new natural comb was then transferred to the outside of the following board in the same hive to facilitate the cleaning of the food stored in the comb by bees. After combs were cleaned, they were taken out of the hive and sent to the laboratory for measurements of cell orientation and cell counts.

### 2.2 Measurements of cell orientation and cell counts

**Fig 2:**
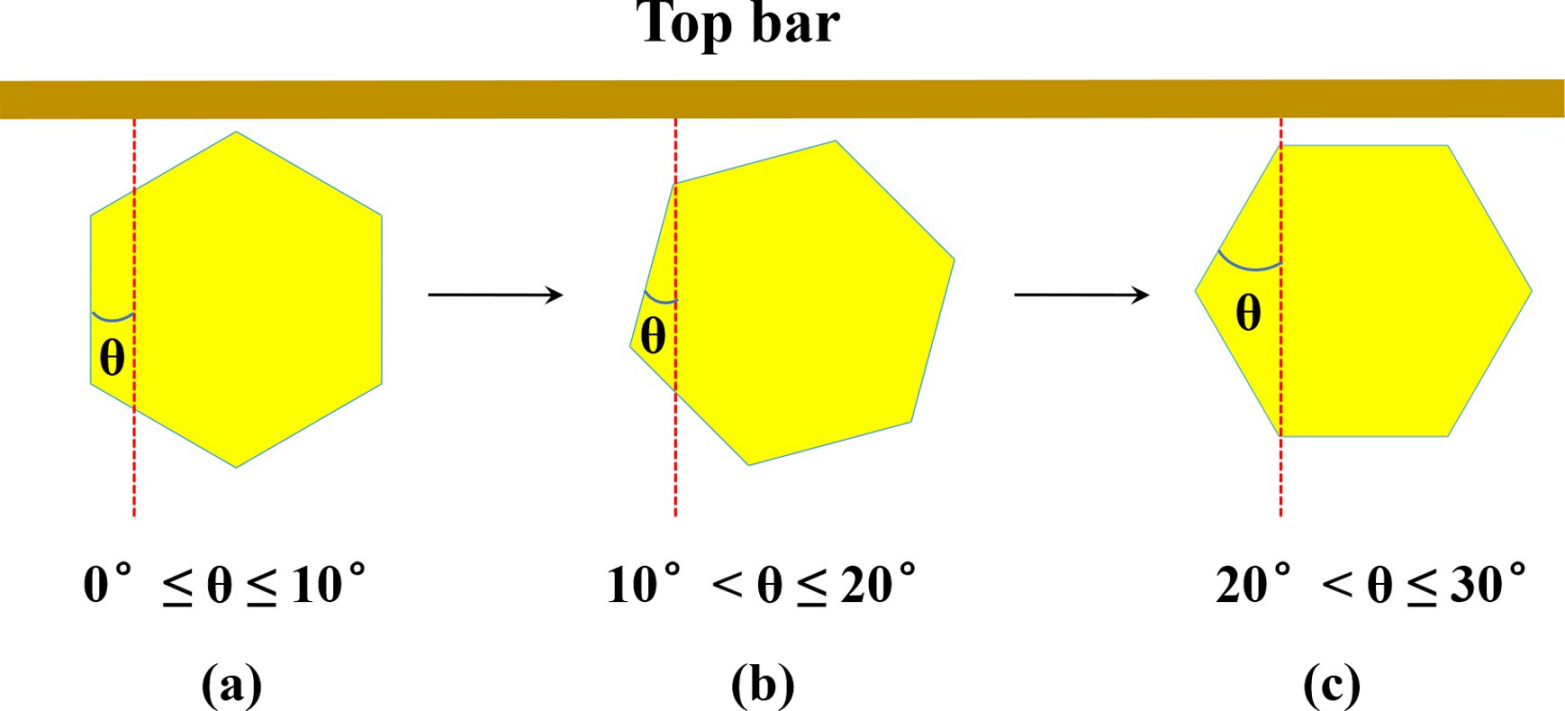
Orientations of the cells, and the minimum included angle between a pair of parallel cell walls and a vertical line connecting the top and bottom bars of the movable frame. Cells oriented in a (a) vertical, (b) intermediate, and (c) horizontal orientation.

In theory, a vertically arranged cell has a pair of parallel cell walls perpendicular to the top and bottom bars of the movable frame. That is, this pair of parallel cell walls is parallel to a vertical line connecting the top and bottom bars of the movable frame in the geometric plane of the comb, and the minimum included angle between the aforementioned pair of parallel cell walls and this vertical line is 0°. Vertically oriented cells become horizontally oriented cells upon rotation 30° clockwise or counterclockwise in the geometric plane of the comb. The minimum angle between the aforementioned pair of parallel walls and a vertical line connecting the top and bottom bars of the movable frame in the geometric plane of the comb is 30°. Therefore, the range of the minimum angle between the aforementioned pair of parallel walls and this vertical line is 0°–30°. According to the classification of the orientation of cells of natural honey bee combs proposed by Shumakova and Komissar [10], cells are oriented vertically when the minimum included angle between a pair of parallel cell walls and a vertical line connecting the top and bottom bars of the movable frame in the geometric plane of the comb is 0°≤θ≤10° (Fig 2A). When this minimum included angle is 10°<θ≤20°, cells are in an intermediate orientation (Fig 2B), and when this minimum included angle is 20°<θ≤30°, the cells are oriented horizontally (Fig 2C). An intermediate orientation of cells can be obtained by rotating vertically or horizontally oriented cells by 10°–20°.

**Fig 3:**
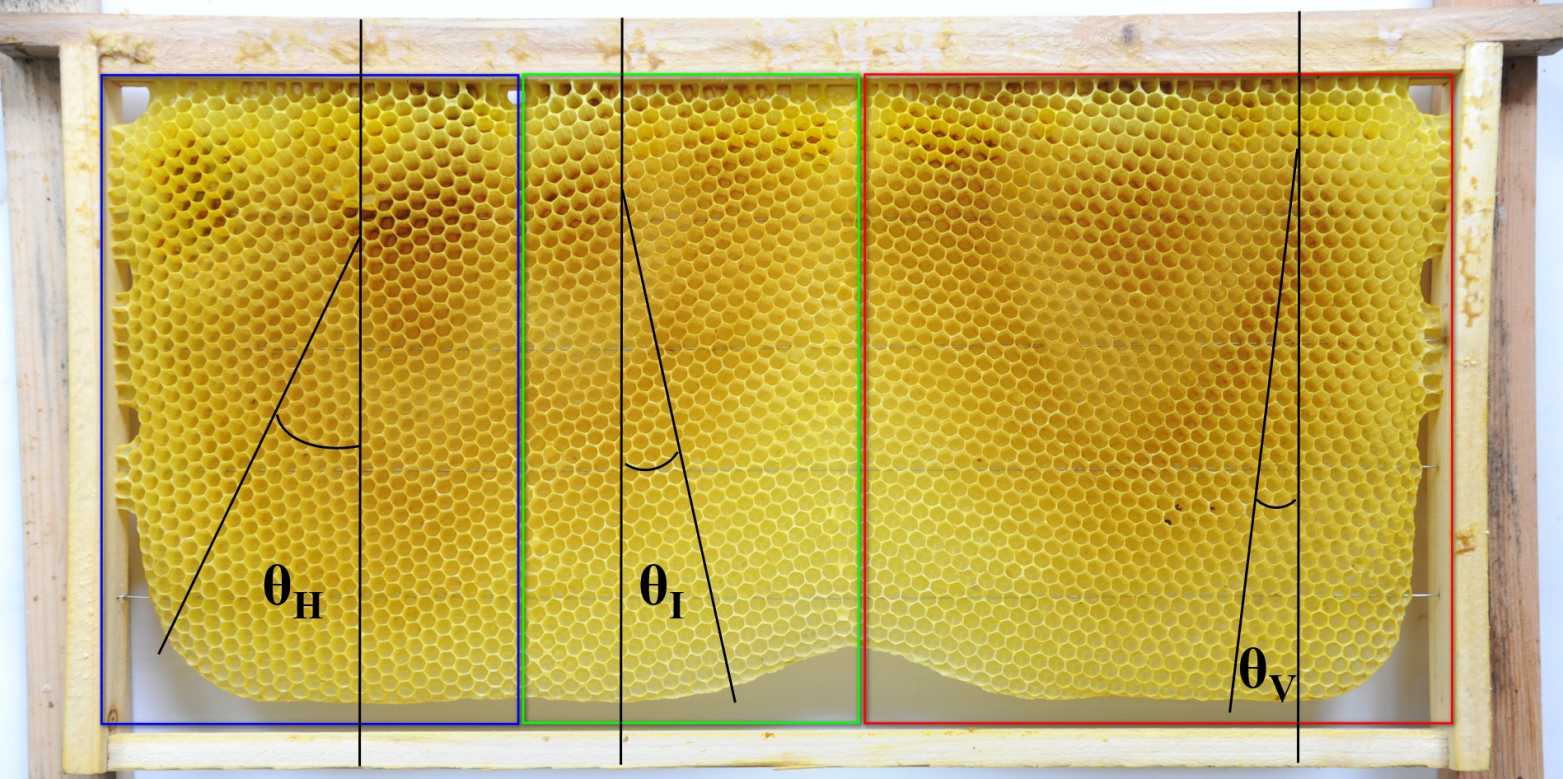
Method for measuring the minimum included angle.

The orientation of cells in natural combs was measured and classified using the above criteria, and θ_**V**_, θ_**I**_, and θ_**H**_ were used to represent the angles of the cells in vertical, intermediate, and horizontal orientations, respectively. Measurement procedures were based on those described in Pratt [16] and Oelsen and Rademacher [9]. The orientation of all cells along a vertical line connecting the top and bottom bars of the movable frame and all cells on both sides of the comb were measured (Fig 3). VN, IN, and HN denote the number of cells in vertical, intermediate, and horizontal orientations, respectively; and N denotes the total number of cells. These data were then used to calculate the proportion of cells in the three orientations. The proportion of cells in a vertical orientation (WV = (VN/N)×100%), the proportion of cells in an intermediate orientation (WI = (IN/N)×100%), and the proportion of cells in a horizontal orientation (WH = (HN/N)×100%) of natural Eastern honey bee and Western honey bee combs were compared.

### 2.3 Oviposition preferences of queen bees for cells in vertical, intermediate, and horizontal orientations

**Fig 4:**
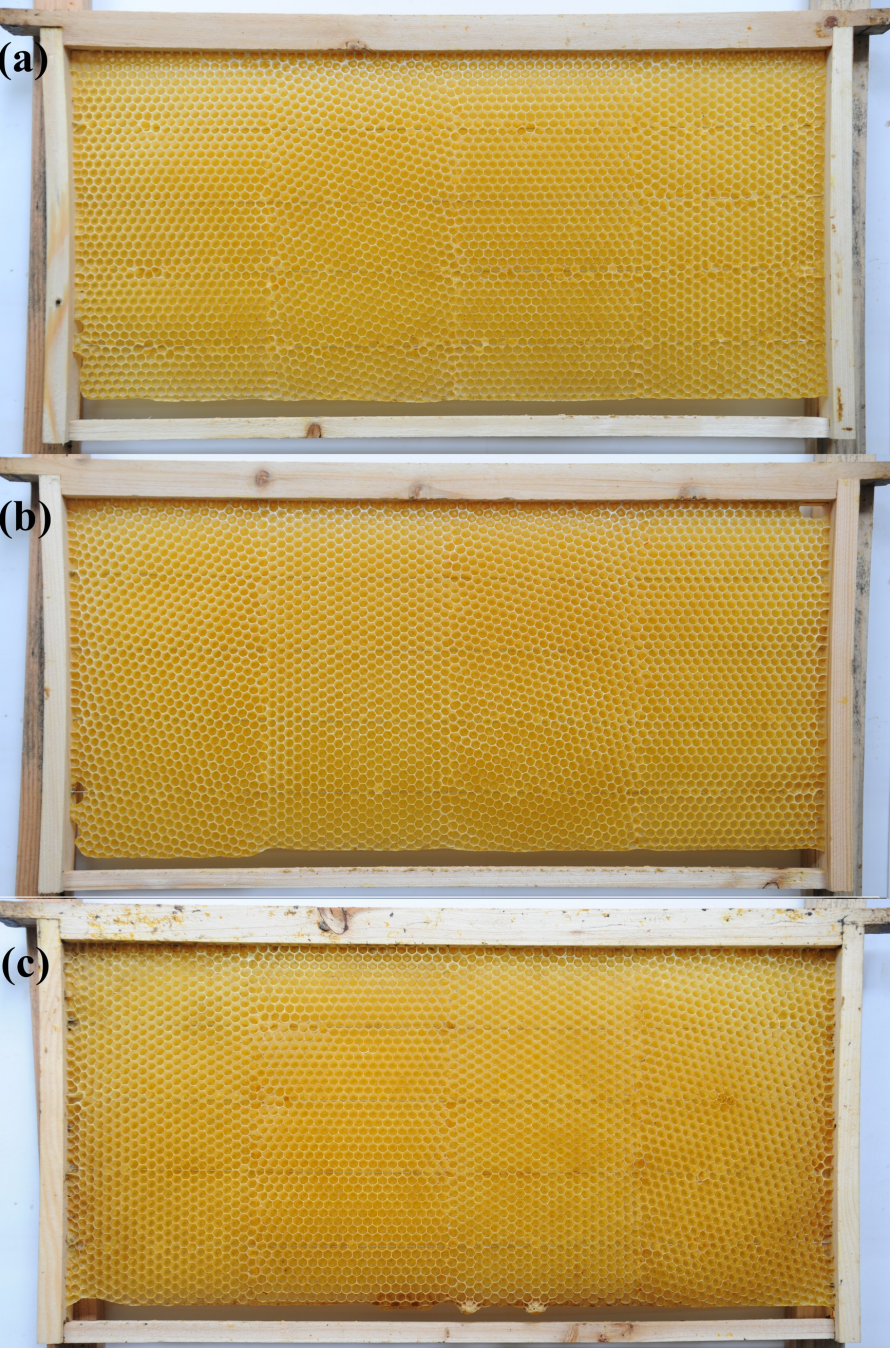
(a) The newly built Western honey bee combs of group A. The installation sequence of the wax comb foundation sheets was vertical, intermediate, vertical, and horizontal for each movable frame; (b) The newly built Western honey bee combs of group B. The installation sequence of the wax comb foundation sheets was intermediate, horizontal, intermediate, and vertical for each movable frame; (c) The newly built Western honey bee combs of group C. The installation sequence of the wax comb foundation sheets was horizontal, vertical, horizontal, and intermediate for each movable frame.

Nine standard movable frames were used to install the wax comb foundations of Western honey bees. Each frame was installed with four pieces of wax comb foundation (length × width: 180 mm × 107 mm). The comb foundation sheets were cut and installed in three ways so that the cell base of the comb foundation had vertical (0°), intermediate (15°), and horizontal (30°) orientations relative to the top bar of the movable frame. The installation sequence of the wax comb foundation sheets on each movable frame is shown in Table 1. In group A, the installation sequence was vertical, intermediate, vertical, and horizontal; in group B, the installation sequence was intermediate, horizontal, intermediate, and vertical; and in group C, the installation sequence was horizontal, vertical, horizontal, and intermediate (Fig 4). Each group was replicated three times. The frames with comb foundation sheets of group A were denoted as A1, A2, and A3; the frames with comb foundation sheets of group B were denoted as B1, B2, and B3; and the frames with comb foundation sheets of group C were denoted as C1, C2, and C3. A, B, and C were then placed into Western honey bee colonies so that they could use them to build new combs. After the above frames were added into the nests, the bee colonies were fed syrup every evening until the construction of new combs was completed. The newly built combs were placed outside the board in the hive, which allowed the bees to easily clean the feed stored in the comb cells. After cleaning, the combs were removed from the hives. The same method described above was used to generate nine newly built Eastern honey bee combs.

**Table 1:** The installation sequence of the wax comb foundation sheets on each movable frame.

Group A	Group B	Group C
**A (A1, A2, A3)**	**B (B1, B2, B3)**	**C (C1, C2, C3)**
V, I, V, H	I, H, I, V	H, V, H, I

Vertical: V; Intermediate: I; Horizontal: H.

Next, the egg-laying preferences of queen bees for cells in different orientations were tested. Three Western honey bee colonies were established in standard movable-frame Langstroth hives. In all three colonies, queens were of the same age, colonies were of similar strength, and six frames were fully covered by adult bees. The newly built combs A1, A2, and A3 and the queens of the above three Western honey bee colonies were placed into the three queen oviposition controllers. The oviposition controllers were placed into colonies with the newly built comb and queen. These combs were removed from the hives after 48 h, and the number of eggs laid by the queen in the cells of the combs was counted. The same methods were used for the combs of group B (B1, B2, and B3) and group C (C1, C2, and C3). The tests were completed over seven to ten days, and all bee colonies were subjected to the same management measures throughout the test period. The same procedures and sample sizes were used to evaluate the egg-laying preferences of Eastern honey bees.

### 2.4 Statistical analysis

All data obtained were analyzed using SAS v8.0 software (SAS Institute Inc., North Carolina, USA). Data were expressed as mean ± standard error. One-way ANOVA was used to compare the proportion of the number of cells in different orientations as well as the number of eggs laid by queens in cells in different orientations (vertical, intermediate, and horizontal). Differences among means were determined by post hoc Tukey’s test. The threshold for statistical significance was *P* < 0.05.

## 3 Results

### 3.1 Orientation of cells of natural honey bee combs

#### 3.1.1 Eastern honey bee worker combs

**Fig 5:**
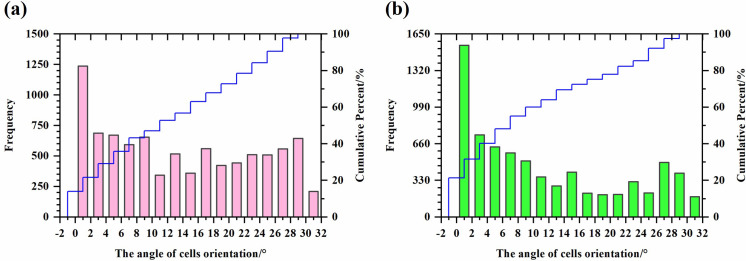
The cell orientation angle of honey bee combs. (a) The frequency distribution histogram of the cell orientation angle of Eastern honey bee (*A*. *c*. *cerana*) combs; (b) The frequency distribution histogram of the cell orientation angle of Western honey bee (*A*. *m*. *ligustica*) combs.

The angle of cells in a vertical orientation was 0.00°≤θ_**V**_≤10.00°, with an average of 4.26°±0.05° (N _cell_ = 3874). The angle of cells in an intermediate orientation was 10.05°≤θ_**I**_≤19.80°, with an average of 15.22°±0.06° (N _cell_ = 2160). The angle of cells in a horizontal orientation was 20.05°≤θ_**H**_≤30.00°, with an average of 25.63°±0.06° (N _cell_ = 2870). The frequency distribution histogram of the cell orientation angle of Eastern honey bee combs is shown in Fig 5A.

#### 3.1.2 Western honey bee worker combs

The angle of cells in a vertical orientation was 0.00°≤θ_**V**_≤10.00°, with an average of 3.69°±0.05° (N _cell_ = 4028). The angle of cells in an intermediate orientation was 10.20°≤θ_**I**_≤20.00°, with an average of 14.81°±0.07° (N _cell_ = 1487). The angle of cells in a horizontal orientation was 20.30°≤θ_**H**_≤30.00°, with an average of 26.21°±0.07° (N _cell_ = 1745). The frequency distribution histogram of the cell orientation angle of Western honey bee combs is shown in Fig 5B.

### 3.2 Proportions of cells in vertical, intermediate, and horizontal orientations

#### 3.2.1 Eastern honey bee worker combs

**Fig 6:**
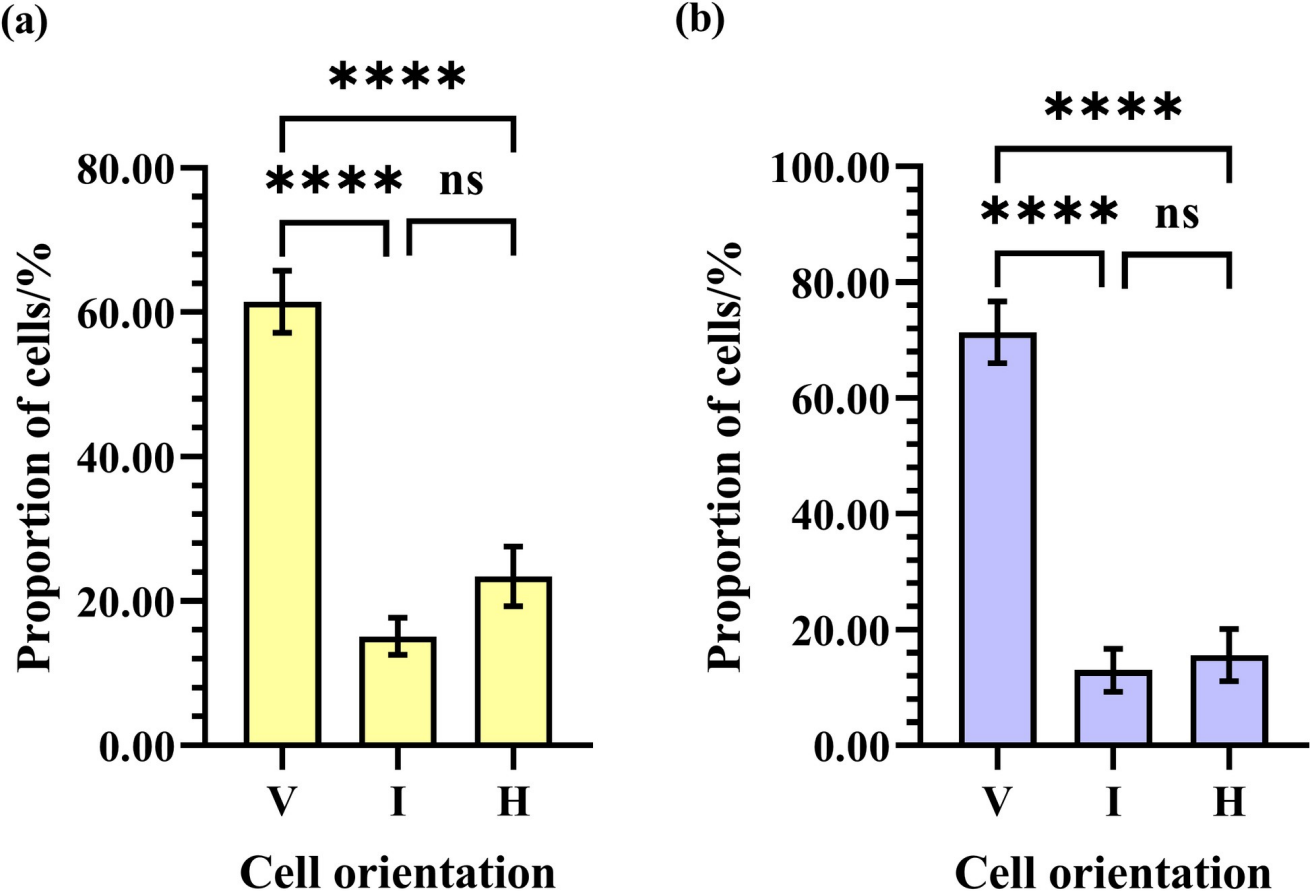
Comparison of the proportions of cells in a vertical (V), intermediate (I), and horizontal (H) orientation in natural (a) Eastern and (b) Western honey bee combs. Asterisks indicate significant differences (*P*<0.05); “ns” indicates differences are not significant (*P*>0.05).

There were significant differences in the proportions of cells in vertical, intermediate, and horizontal orientations (F = 43.43; df = 2, 111; *P*<0.0001). WV significantly differed from WI and WH (*P*<0.0001). There was no significant difference between WI and WH (*P* = 0.2648). The average values of WV, WI, and WH were 61.46%±4.30% (N _cell_ = 238897), 15.12%±2.55% (N _cell_ = 238897), and 23.42%±4.15% (N _cell_ = 238897), respectively (Fig 6A).

#### 3.2.2 Western honey bee worker combs

There were significant differences in the proportions of cells in vertical, intermediate, and horizontal orientations (F = 52.41; df = 2, 78; *P*<0.0001). WV significantly differed from WI and WH (*P* <0.0001). There was no significant difference between WI and WH (*P* = 0.9166). The average values of WV, WI, and WH were 71.39%±5.32% (N _cell_ = 146719), 13.02%±3.70% (N _cell_ = 146719), and 15.59%±4.50% (N _cell_ = 146719), respectively (Fig 6B).

### 3.3 Proportion of combs with cells in different orientations

#### 3.3.1 Eastern honey bee worker combs

**Fig 7:**
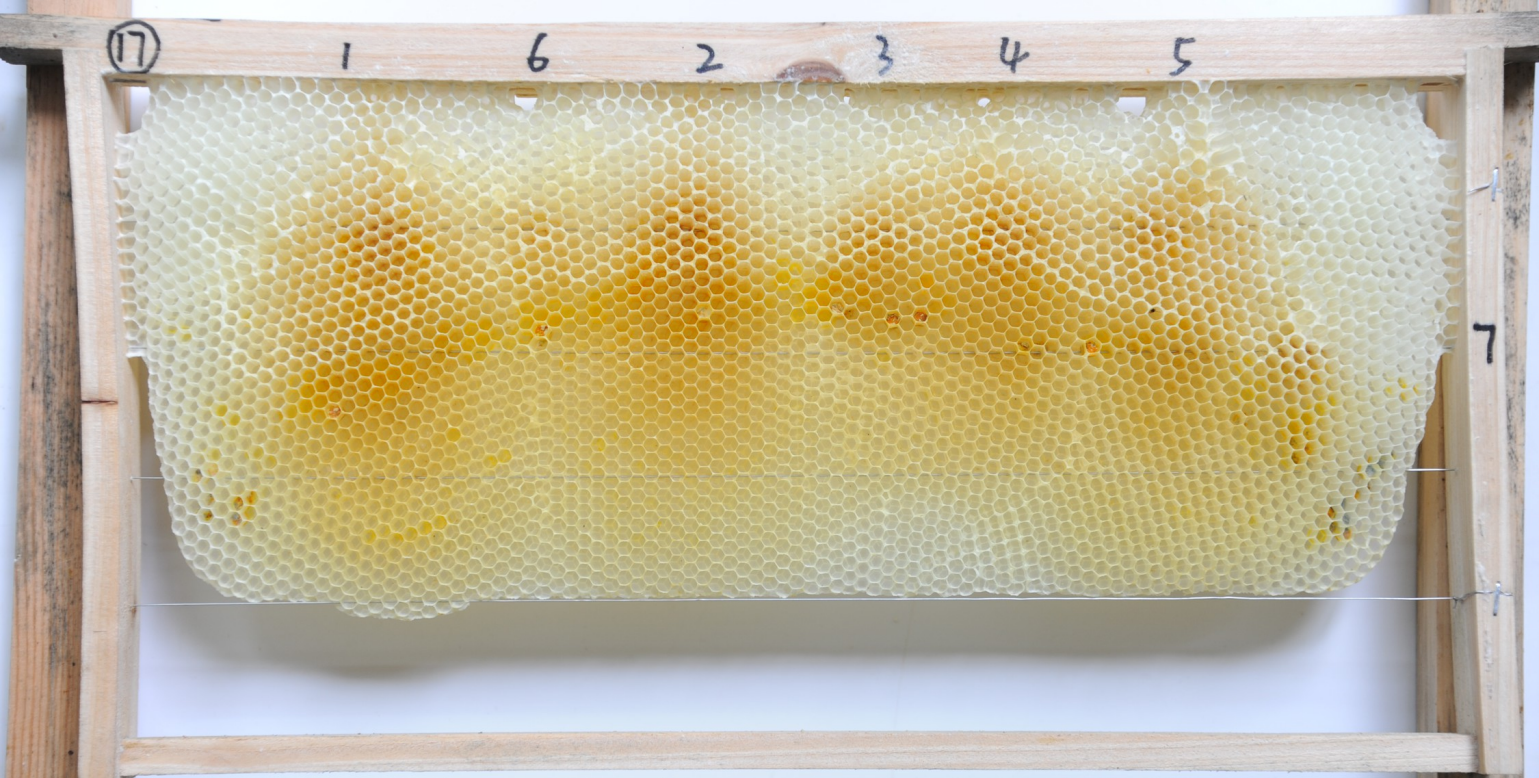
Natural Eastern honey bee comb with cells in a horizontal orientation.

**Fig 8:**
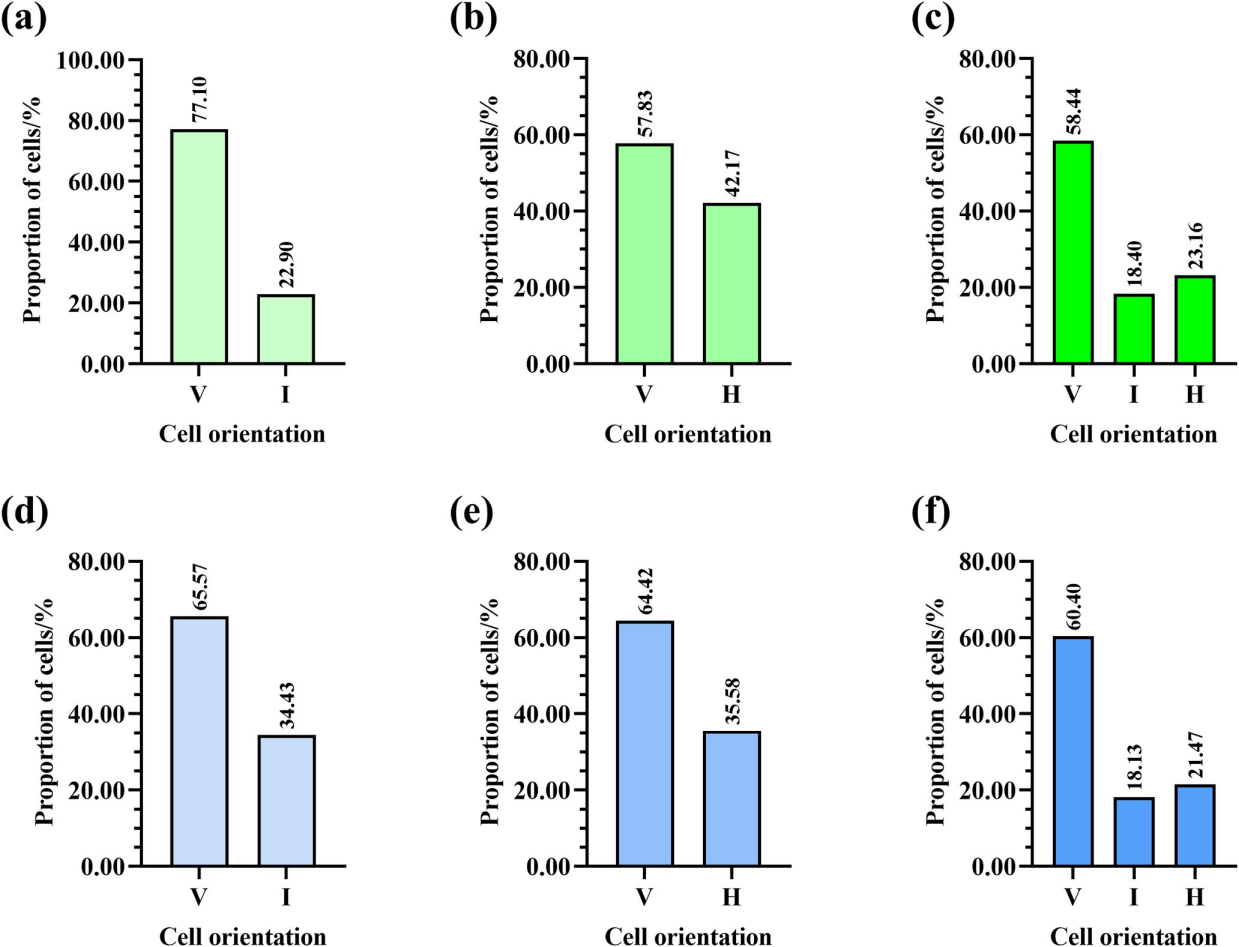
Proportions of cells in different orientations in natural Eastern honey bee combs with (a) cells in vertical (V) and intermediate (I) orientations, (b) cells in V and horizontal (H) orientations, and (c) cells in V, I, and H orientations. Proportions of cells in different orientations in natural Western honey bee combs with (d) cells in vertical (V) and intermediate (I) orientations, (e) cells in V and horizontal (H) orientations, and (f) cells in V, I, and H orientations.

**Fig 9:**
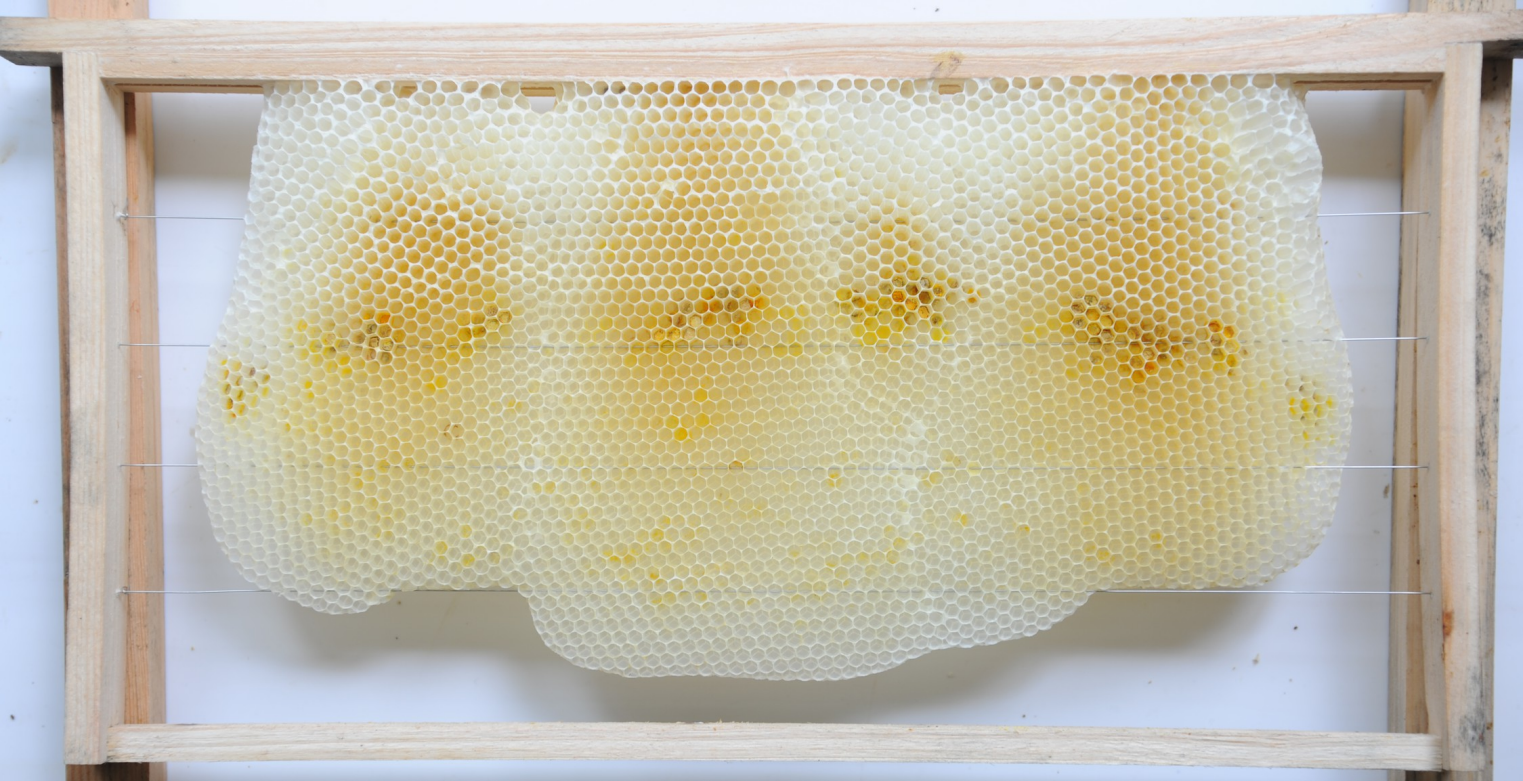
Natural Eastern honey bee comb with cells in vertical and horizontal orientations.

**Fig 10:**
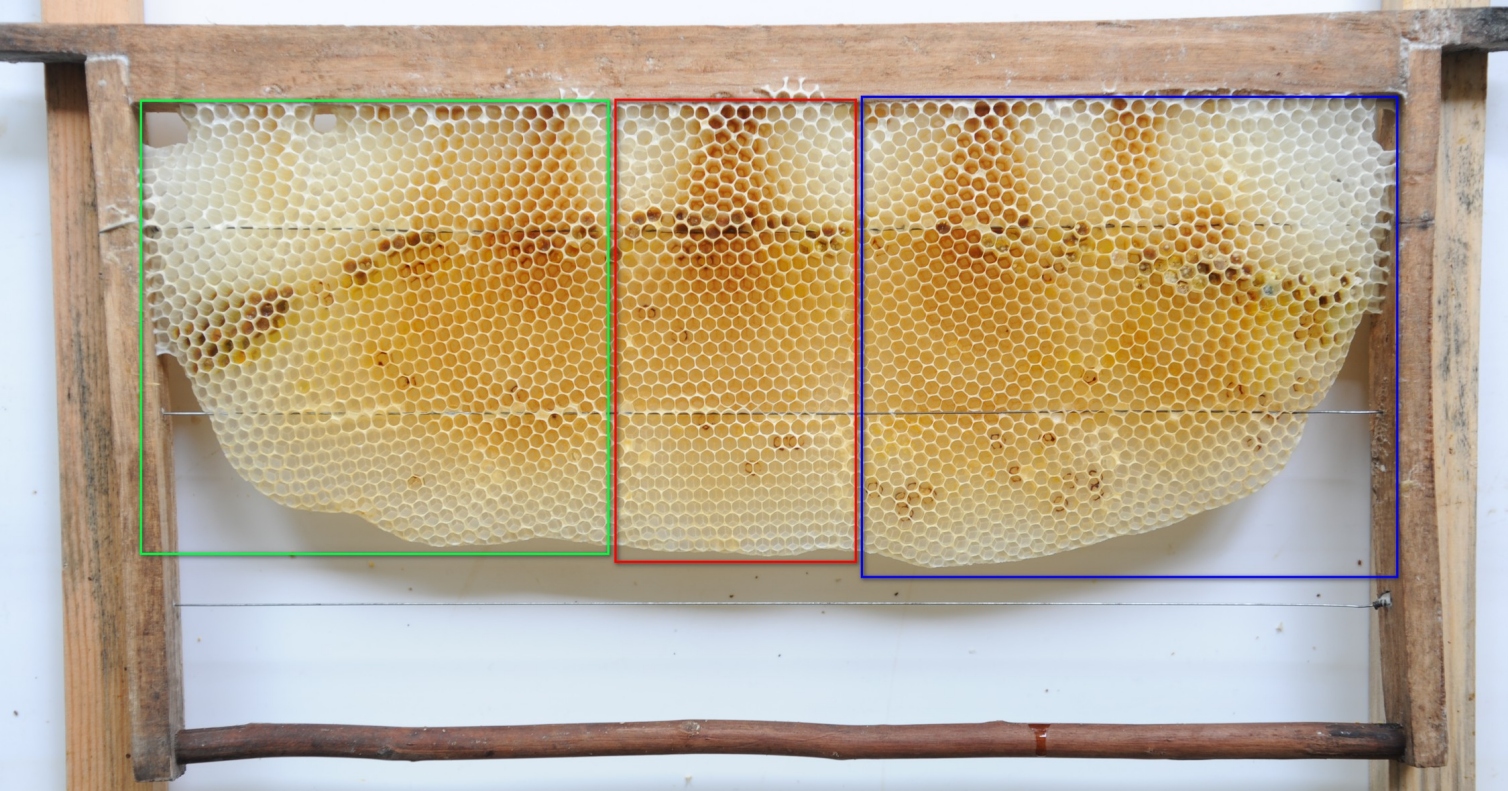
Natural Eastern honey bee combs with cells in vertical (outlined in red), intermediate (outlined in green), and horizontal (outlined in blue) orientations.

Two combs (5.26%) had cells in a vertical orientation (N _cell_ = 12994). Only one comb (2.63%) had cells in a horizontal orientation ((N _cell_ = 6090, Fig 7). Five combs (13.16%) had cells in vertical and intermediate orientations (**Table 2**), and the proportions of cells in vertical and intermediate orientations in these five combs were 77.10% (N _cell_ = 29604) and 22.90% (N _cell_ = 29604), respectively (Fig 8A). Five combs (13.16%) had cells in vertical and horizontal orientations (**Table 2** and Fig 9), and the proportions of cells in vertical and horizontal orientations in these five combs were 57.83% (N _cell_ = 33375) and 42.17% (N _cell_ = 33375), respectively (Fig 8B). Twenty-five combs (65.79%) had cells in vertical, intermediate, and horizontal orientations. The proportions of cells in vertical, intermediate, and horizontal orientations (Fig 10) in these 25 combs were 58.44% (N _cell_ = 156834), 18.40% (N _cell_ = 156834), and 23.16% (N _cell_ = 156834), respectively (Fig 8C).

**Table 2:** Number and proportion (%) of Eastern honey bee combs (n = 38) with cells in different orientations (vertical: V; intermediate: I; horizontal: H).

Orientation type	V	I	H	V + I	V + H	I + H	V + I + H
Number	2	0	1	5	5	0	25
Proportion	5.26%	0.00%	2.63%	13.16%	13.16%	0.00%	65.79%

#### 3.3.2 Western honey bee worker combs

**Fig 11:**
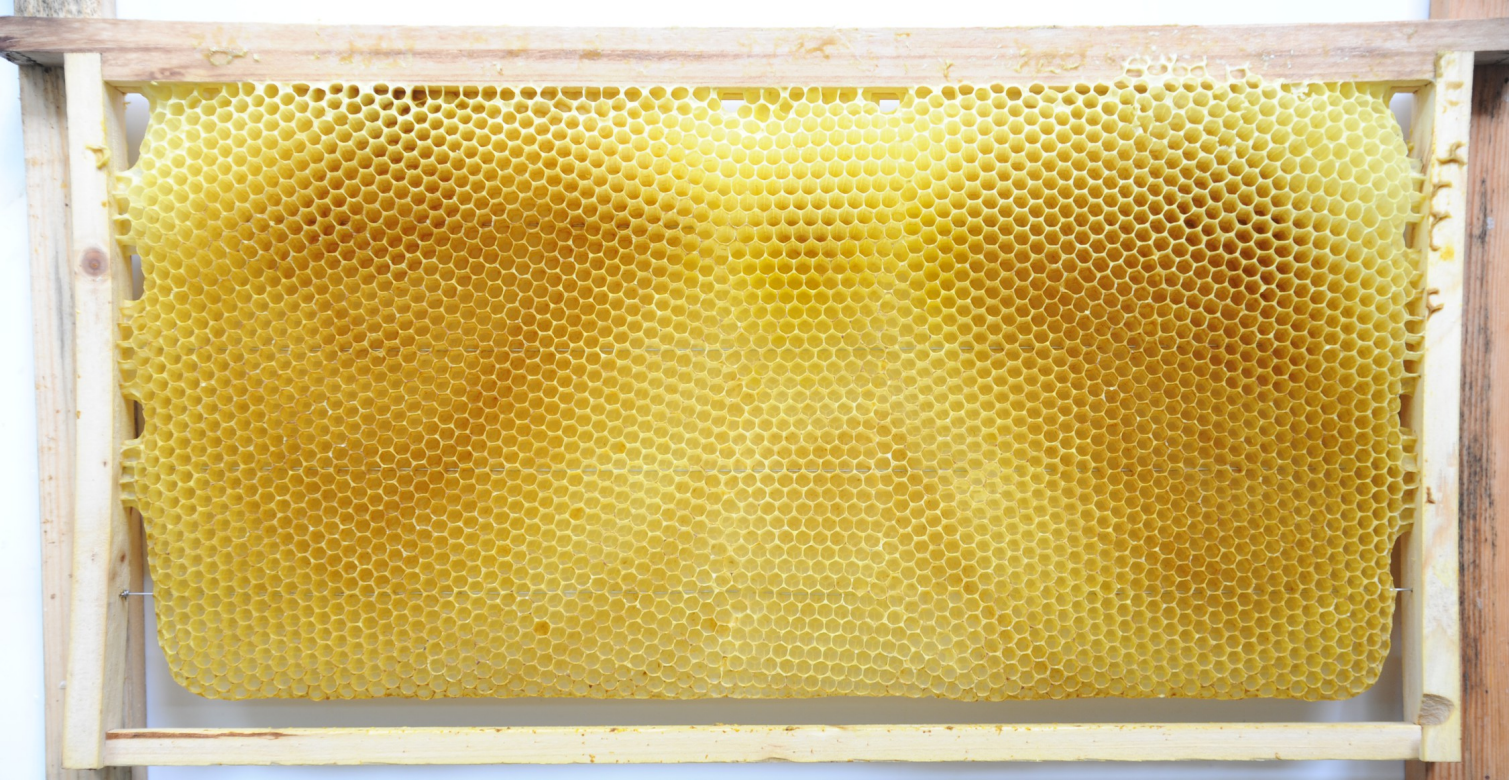
Natural Western honey bee comb with cells in vertical and horizontal orientations.

**Fig 12:**
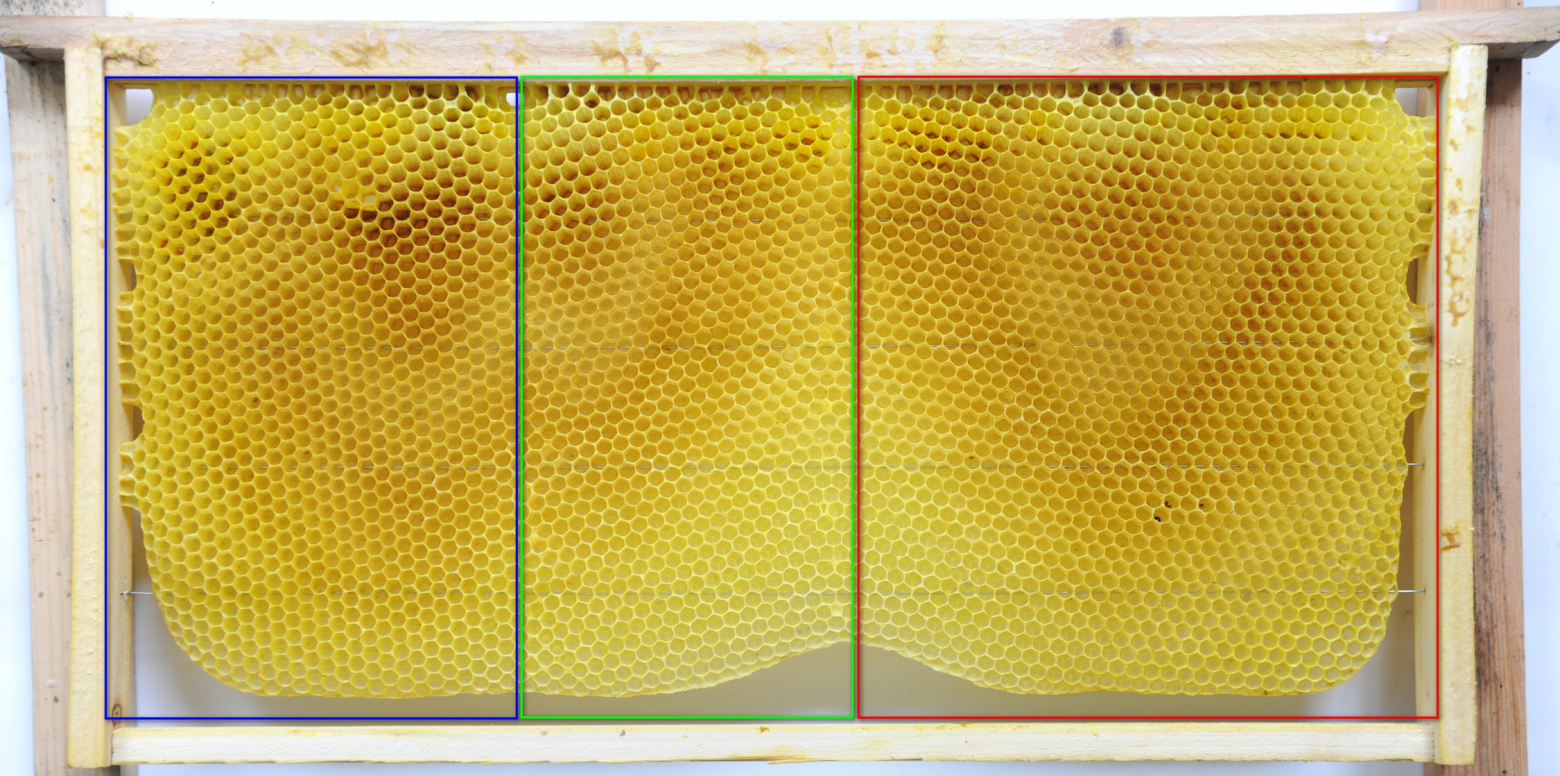
Natural Western honey bee comb with cells in vertical (outlined in red), intermediate (outline in green), and horizontal (outlined in blue) orientations.

Six combs (22.22%) had cells in a vertical orientation (N _cell_ = 31333). Six combs (22.22%) had cells in vertical and intermediate orientations (**Table 3**), and the proportions of cells in vertical and intermediate orientations in these six combs were 65.57% (N _cell_ = 31347) and 34.43% (N _cell_ = 31347), respectively (Fig 8D). Seven combs (25.93%) had cells in vertical and horizontal orientations (**Table 3** and Fig 11), and the percentages of cells in vertical and horizontal orientations in these seven combs were 64.42% (N _cell_ = 39140) and 35.58% (N _cell_ = 39140), respectively (Fig 8E). Eight combs (29.63%) had cells in vertical, intermediate, and horizontal orientations (Fig 12). The proportions of cells in vertical, intermediate, and horizontal orientations in these eight combs were 60.40% (N _cell_ = 44899), 18.13% (N _cell_ = 44899), and 21.47% (N _cell_ = 44899), respectively (Fig 8F).

**Table 3:** Number and proportion of Western honey bee combs (n = 27) with cells in different orientations (vertical: V; intermediate: I; horizontal: H).

Orientation type	V	I	H	V + I	V + H	I + H	V + I + H
Number	6	0	0	6	7	0	8
Proportion	22.22%	0.00%	0.00%	22.22%	25.93%	0.00%	29.63%

### 3.4 Proportion of cells in a rosette arrangement

#### 3.4.1 Eastern honey bee worker combs

Three (7.89%) of the 38 natural Eastern honey bee combs had cells in a rosette arrangement. The bottom of the cells in combs with cells in a rosette arrangement was made up of four or more planes. Among these three combs with cells in a rosette arrangement, only one comb had two rosette arrangements: vertical + intermediate and horizontal + intermediate. The other two combs had only one rosette arrangement: vertical + horizontal and vertical + intermediate.

#### 3.4.2 Western honey bee worker combs

**Fig 13:**
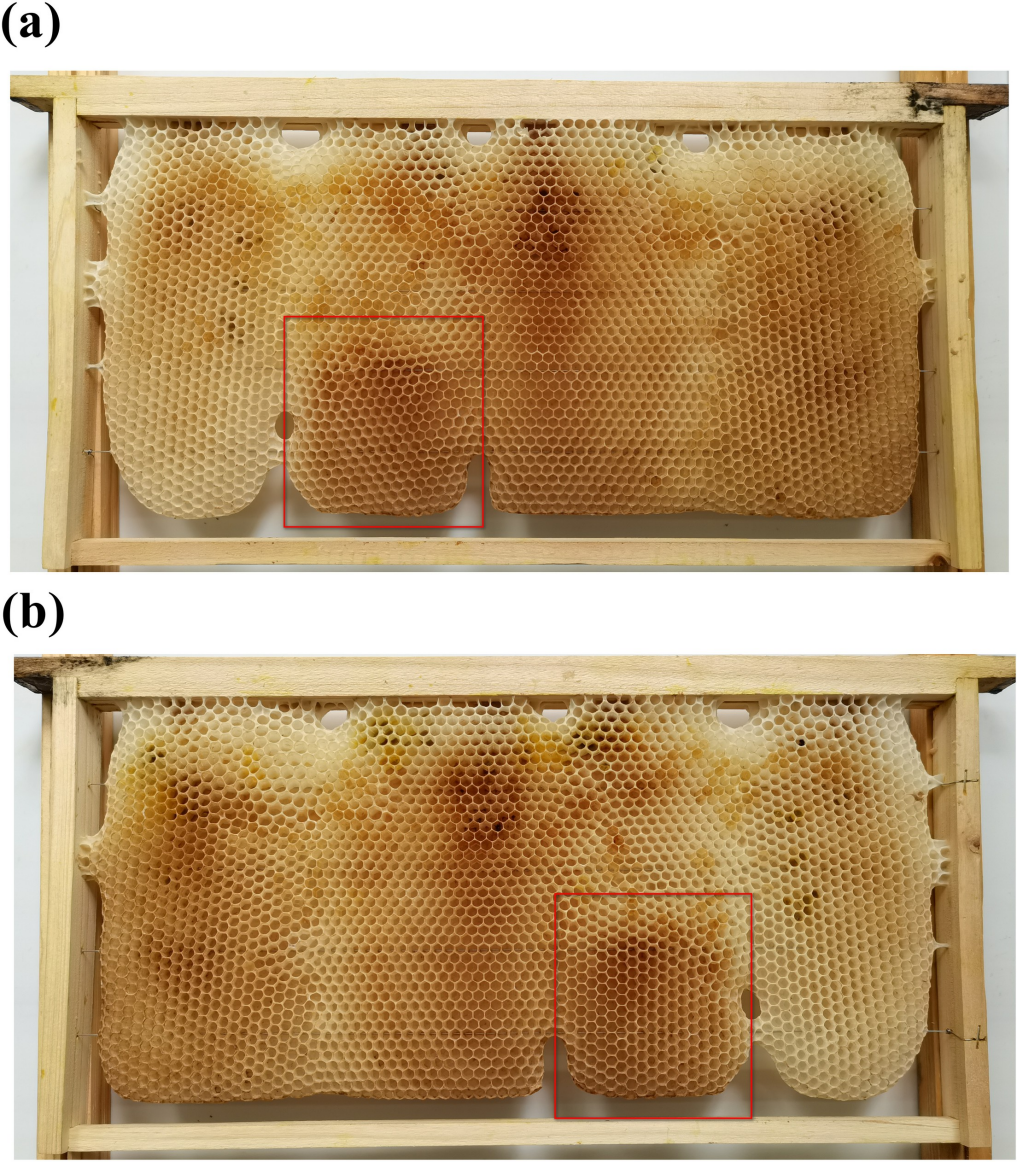
Natural Western honey bee combs with cells in a rosette arrangement. (a) Cells enclosed by the red rectangle are in a vertical orientation; (b) cells enclosed by the red rectangle are in a horizontal orientation.

Three of the 27 (11.11%) natural Western honey bee combs had cells in a rosette arrangement. The bottom of the cells in combs with cells in a rosette arrangement was made up of four or more planes. Among the three combs with cells in a rosette arrangement, only one had two rosette arrangements: vertical + intermediate and vertical + horizontal. The other two combs had only one rosette arrangement: vertical + horizontal (Fig 13) and vertical + intermediate.

### 3.5 Oviposition preferences of queen bees for comb cells in different orientations

#### 3.5.1 Eastern honey bees

**Fig 14:**
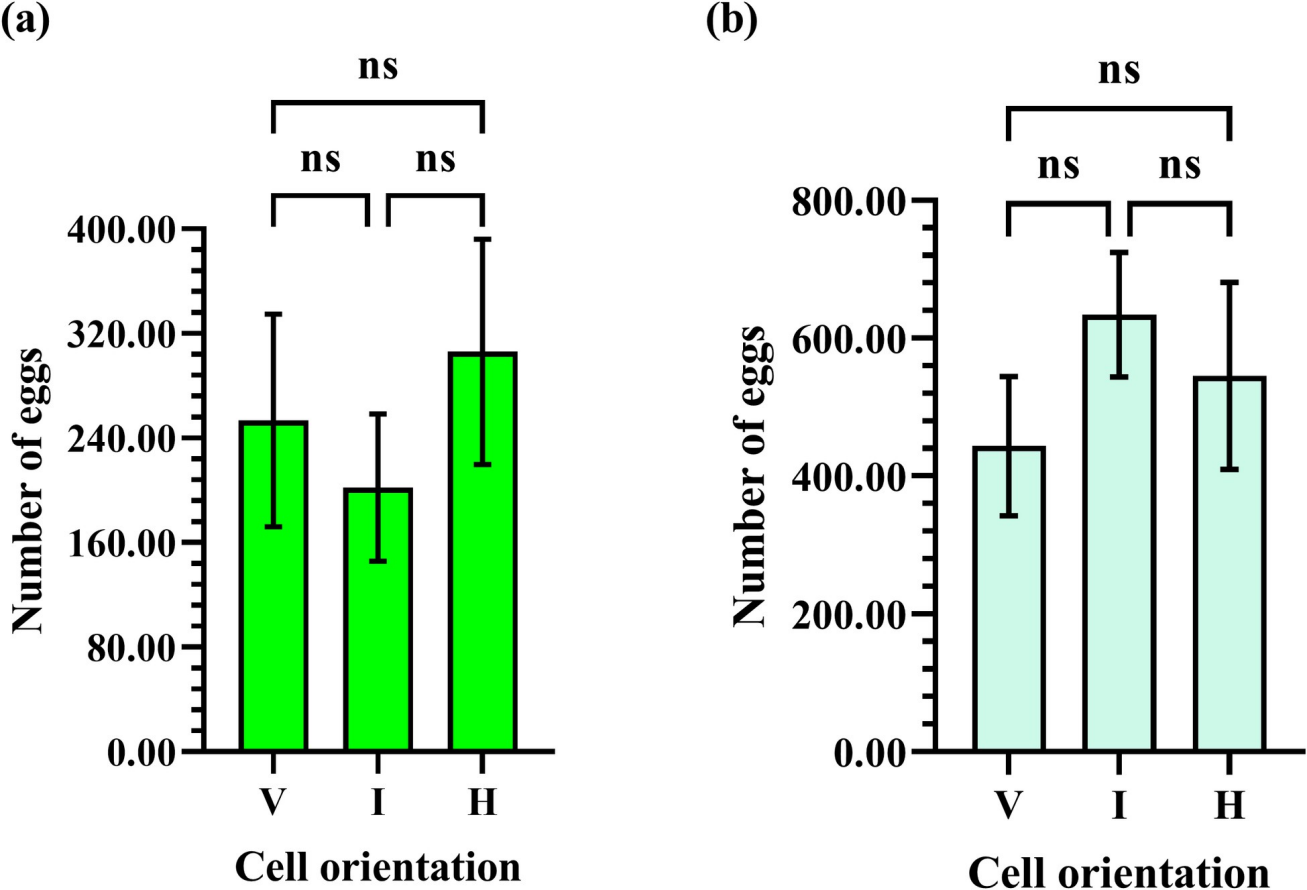
Number of eggs laid by (a) Eastern honey bee queens and (b) Western honey bee queens in cells in vertical (V), intermediate (I), and horizontal (H) orientations. The acronym “ns” indicates that differences are not significant (*P*>0.05).

There was no significant difference in the number of eggs laid by Eastern honey bee queens in cells in vertical, intermediate, and horizontal orientations (F = 0.4715; df = 2, 33; *P* = 0.6282). The average number of eggs laid in cells in vertical, intermediate, and horizontal orientations was 253.5± 81.19 (N _egg_ = 9140), 202.2±56.23 (N _egg_ = 9140), and 306.0±86.01 (N _egg_ = 9140), respectively (Fig 14A).

#### 3.5.2 Western honey bees

There was no significant difference in the number of eggs laid by Western honey bee queens in cells in vertical, intermediate, and horizontal orientations (F = 0.7403; df = 2, 33; *P* = 0.4847). The average number of eggs laid in cells in vertical, intermediate, and horizontal orientations was 443.6±101.0 (N _egg_ = 19475), 634.0±90.01 (N _egg_ = 19475), and 545.3±136.0 (N _egg_ = 19475), respectively (Fig 14B).

## 4 Discussion

### 4.1 Classification of the orientation of cells in natural honey bee combs

In most cases, the combs of the natural nests of Eastern honey bees and Western honey bees hang vertically on the top of the cavity, and the cells are regular hexagons with three pairs of parallel cell walls. Early researchers classified the orientation of natural cells into vertical, horizontal, and intermediate. In a vertical orientation, each regular hexagonal cell has a pair of parallel cell walls oriented perpendicular to the top and bottom bars of the movable frame, and the minimum included angle between the pair of parallel cell walls and a vertical line connecting the top and bottom bars of the movable frame in the geometric plane of the comb is 0°. In a horizontal orientation, each regular hexagonal cell has a pair of parallel cell walls oriented parallel to the top and bottom bars of the movable frame, and the minimum included angle between the other two pairs of parallel cell walls and a vertical line connecting the top and bottom bars of the movable frame in the geometric plane of the comb is 30°. In an intermediate orientation, each regular hexagonal cell has a pair of parallel cell walls that is oriented neither perpendicular nor parallel to the top and bottom bars of the movable frame, and the minimum included angle between the pair of parallel cell walls and a vertical line connecting the top and bottom bars of the movable frame in the geometric plane of the comb is 0°<θ<30° 0) [5, 6]. Rotation of a regular hexagon by 60° clockwise or counterclockwise in the geometric plane can return it to its original position, and horizontally oriented regular hexagonal cells can be converted into vertically oriented cells via rotation by 30° clockwise or counterclockwise in the geometric plane. Therefore, 0°–30° can be used to indicate changes in cell orientation. In natural combs, cells are rarely arranged in a completely vertical (0°) or horizontal (30°) orientation. Therefore, some researchers have proposed an alternative approach for classifying the orientation of cells of natural honey bee combs. Shumakova and Komissar [10] considered cells showing deviations from horizontal and vertical lines of ±10° to be in either vertical or horizontal orientations and cells within 10°–20° to be in intermediate orientation. This latter classification scheme was used in this study to characterize the orientation of the cells of natural combs of Eastern honey bees and Western honey bees.

### 4.2 Oviposition preferences of queens for cells in different orientations

At the colony level, Eastern honey bees and Western honey bees prefer to build cells in a vertical orientation. Honey bees are thought to prefer building cells in a vertical orientation because the strength of combs with vertically oriented cells is thought to be greater than the strength of combs with cells in an intermediate or horizontal orientation. However, there is a lack of consensus in the literature regarding the relationship between comb strength and cell orientation. Wedmore [17] showed that combs with cells in a vertical orientation did not differ in strength compared with combs with cells in a horizontal orientation. However, Betts [18] suggested that the bearing capacity and thus strength of the comb are affected by cell orientation. Other researchers have examined this issue from the perspective of the wax comb foundation. The strength of the wax comb foundation is greater when the cell base is in a horizontal orientation compared with a vertical orientation [19, 20]. However, simulations of aluminum honeycombs have shown that the strength of aluminum honeycombs with cells in a vertical orientation is higher than that of aluminum honeycombs with cells in a horizontal orientation [21]. Thus, whether honey bees prefer to build natural combs with cells in a vertical orientation because such combs are stronger remains unclear.

At the individual level, queens show no clear preference for laying eggs in cells of specific orientations. Nevertheless, we speculate that the queens typically laid eggs in cells corresponding to the middle two pieces of the wax comb foundation sheets of combs of groups A, B, and C. Previous work has shown that the brood area is distributed in the central area of the comb, the pollen surrounds the brood area, and the honey is distributed at the edges of the comb [22–25], which suggests that queens tend to preferentially lay eggs in the central area of the comb.

The results of our study suggest that beekeepers of Eastern honey bees and Western honey bees should install the wax comb foundation into the movable frame with cells in a vertical orientation because this is closer to the preferences of honey bees at the colony level. In addition, wax comb foundation manufacturers should ensure that comb foundations are installed into the movable frame with the cell base in a vertical orientation, which is in line with the preferences of Eastern and Western honey bees. Although additional work is needed to determine the effect of installing the wax comb foundation in a vertical orientation for Eastern honey bee and Western honey bee apiculture, the results of our research indicate that installing the comb foundation in an orientation in line with the comb-building preferences at the colony level could only have a beneficial effect. We plan to conduct future studies to identify the orientation most beneficial to Eastern honey bee and Western honey bee apiculture.

### 4.3 Variation in cell orientation and the mechanism of honey bee comb construction

Natural combs with cells in vertical, intermediate, and horizontal orientations in the movable frame can be constructed by domestic honey bees nesting in the hive [6, 10]. Natural colonies in the wild can also build natural combs with cells in vertical, intermediate, and horizontal orientations. For example, previous studies have shown that Western honey bees in the wild built natural combs with cells in intermediate [22], vertical, and horizontal orientations [26]. Cape honey bees (*Apis mellifera capensis*) (Hymenoptera: Apidae) have been noted to construct natural combs with cells in vertical and intermediate orientations [27].

When honey bees build natural combs in movable frames, the first row of cells is often placed at the top bar. The first row of cells provides the foundation for building the second row of cells, and each row of cells built thereafter serves as the foundation for the construction of subsequent rows. Thus, combs are built through the continuous accumulation of rows of cells [28]. We found that the shape of the cells in the first cell row on the top bar of the movable frame, which can be quadrilateral, pentagon, or hexagon in shape, had a substantial effect on the orientation of subsequent rows of cells. When the first row of cells attached to the top bar of the movable frame and the second row of cells are in a horizontal orientation, the cells of subsequent rows are built in a horizontal orientation. In the first row of polygonal cells, when all cell walls are inclined relative to the top bar of the movable frame and the opening size varies greatly, the subsequent cells are built in an intermediate orientation. When the size of the first row of polygonal (pentagonal) cells attached to the top bar of the movable frame does not vary, and there is a pair of parallel cell walls perpendicular to the top bar, the subsequent cells are built in a vertical orientation. The insertion of a polygonal cell at any position on the comb may mediate the conversion between cells in vertical and horizontal orientations. Honey bees build natural combs in a cave or on the same movable frame, and they always build several pieces of small combs with cells in different orientations in several locations at the same time. As construction of the comb progresses, the small pieces of combs are merged into a large piece of comb, and the polygonal cells provide the bridge needed for the fusion of small pieces of combs [27, 29, 30]. Natural combs are double-sided structures, and the misalignment of cells on both sides of the comb is rare. Misalignments might be caused by mistakes during the construction process.

Some researchers have suggested that the process by which bees construct combs might be explained by the stigmergy hypothesis, which holds that appropriate building behavior is stimulated by previous construction [29, 31–33]. In other words, each builder works according to an algorithm that tells the builder what to do upon encountering a structure at a particular stage of development. The actions of builders transform the original structure into a new structure, and the new structure in turn stimulates the builder and other builders to repeat the action until the structure is completed. According to stigmergy theory, previously constructed cells stimulate and guide workers to build subsequent cells [34]. Several individual worker bees participate in the construction process, and the workers alter the shape of the cells and their orientation [33, 35, 36]. A recently proposed alternative mechanism of honey bee comb construction is the attachment-excavation model [37]. In this model, there are two types of workers: attachers who secrete and attach wax and excavators who excise the attached wax. Worker bees may perform both of these roles. As excavators rely on their antennae to identify the position and amount of beeswax attached, worker bees can excavate beeswax in complete darkness. The cooperation between the attachers and excavators eventually leads to the formation of honey bee comb cells, and this process of comb construction is very rapid. We speculate that attachers or excavators may alter the shape of the cells, which can potentially lead to the formation of cells in different orientations.

## 5 Conclusions

The natural combs of worker bees constructed by Eastern honey bees and Western honey bees contain cells in either vertical, horizontal, both vertical and horizontal, or vertical, intermediate, and horizontal orientations. Both Eastern and Western honey bees prefer building cells in a vertical orientation. Queens show no preference for laying eggs in cells with specific orientations. We suggest that beekeepers of Eastern and Western honey bees install wax foundations into movable frames with the cell base in a vertical orientation given that this is the most common orientation of cells in their combs. Similarly, wax foundation manufacturers should produce wax foundations with the cell base in a vertical orientation.
